# How personality traits of neuroticism and extroversion predict the effects of the COVID-19 on the mental health of Canadians

**DOI:** 10.1371/journal.pone.0251097

**Published:** 2021-05-19

**Authors:** Anahita Shokrkon, Elena Nicoladis

**Affiliations:** Department of Psychology, University of Alberta, Edmonton, AB, Canada; Aalborg University, DENMARK

## Abstract

The Coronavirus Disease (COVID-19) epidemic was first detected in China in December 2019 and spread to other countries fast. Some studies have found that COVID-19 pandemic has had adverse mental health consequences. Individual differences such as personality could contribute to people’s behaviors during a pandemic. In the current study, we examine how personality traits of neuroticism and extroversion (using the Five-Factor Model as our framework) are related to the mental health of Canadians during the COVID-19 pandemic. Using data from an online survey with 1096 responses, this study performed multiple regression analysis to explore how personality traits of neuroticism and extroversion predict the effects of COVID-19 on the mental health of Canadians. The results showed that personality traits of neuroticism and extroversion are associated with the current mental health of Canadians during COVID-19 pandemic, with extroversion positively related to mental health and neuroticism negatively related to it. Results contribute to the management of individual responses to the COVID-19 pandemic and could help public health services provide personality-appropriate mental health services during this pandemic.

## Introduction

The Coronavirus Disease (COVID-19) epidemic was first detected in China in December 2019 and spread to other countries, including Canada, in early 2020. In Canada, the first case of respiratory infection by COVID-19 was reported on 23 January 2020 [1]. The World Health Organization (WHO) classified the COVID-19 outbreak as a pandemic in March 2020 (WHO 2020a) and as a result, in many countries including Canada, serious public restriction measures were announced. For instance, in March 2020, a state of emergency was declared in Canada, travel restrictions were announced and non-essential businesses closure mandated [2, 3]. Previous outbreaks, such as the outbreak of Severe Acute Respiratory Syndrome (SARS) in 2003 and Ebola in 2014 showed that social restrictions had great and long-lasting negative effects on mental health of people and the consequences included acute stress disorders, anxiety, irritability, poor concentration, deteriorating work performance, post-traumatic stress disorders, high psychological distress, depressive symptoms and insomnia [4]. The new COVID-19 disease and the consequences including worries over health, financial issues, job losses, working from home, home-schooling of children, lack of physical contact with other family members, friends and colleagues, and unpredictability have also caused severe psychological stress for many individuals around the world according to researchers [5, 6] and international organizations such as WHO [7].

However, some individuals experienced the psychological effects of COVID-19-related life changes more severely than the others [8]. The results of a poll in April 2020 by Angus Reid Institute showed that while 40% of Canadians reported that their mental health has worsened, another 10% reported that their mental health had worsened “a lot” [9]. Angus Reid Institute later published another study showing that as many Canadians have become more isolated, they announced that they are more worried about their mental health. Their results showed that, in 2019, two-third (67%) of Canadians rated their mental health as good or very good but this year just 53% said the same. One-in-five (19%) now rated their mental health as either poor or very poor [10].

Individual differences in characteristic patterns of thinking, feelings, behaviours, and emotions could contribute to people’s behaviors during a pandemic. For instance, individual differences (such as personality traits) could significantly impact risk perception of a pandemic [11]. Also, in non-pandemic times, people who score higher in extroversion trait, tend to have greater mental health and neurotics do not [13–17, 19], and with the pandemic-related social isolation, this pattern could change. The present study tested how personality traits of neuroticism and extroversion, from the Five-Factor Model (FFM), are related to the mental health of Canadians during the COVID-19 pandemic. For the sake of coherence and in order to address our research hypothesis, in the present study we focused only on personality traits of neuroticism and extroversion. The FFM is the most well-known model of personality consisting of the five broad domains of neuroticism (or emotional instability vs. stability), extroversion (vs. introversion), openness to experience (or unconventionality), agreeableness (vs. antagonism), and conscientiousness (or constraint vs. disinhibition) [12]. Understanding how personality contributes to people’s mental health status during the COVID‐19 pandemic could provide guidance for public health services to develop personality‐tailored advice and help construct evidence-based recommendations for future infectious disease outbreaks. I will elaborate more on the connection between personality and mental health below.

In pre-pandemic time, out of the five-factor model of personality (FFM), neuroticism and extroversion tend to show the strongest links to mental health [13–17, 19]. People who score high on the neuroticism trait usually experience more negative affectivity (i.e. anxiety, anger, self‐consciousness, irritability, and fear), respond worse to stressors leaving them vulnerable to adverse outcomes in the context of stressful experiences, are more anxious and insecure [18], predisposing them to psychological distress [19], and act more impulsively in comparison to individuals who score low on neuroticism [20]. High neuroticism is associated with a range of detrimental health outcomes, including lower subjective well-being [21], depressive symptoms, anxiety, mood, and substance abuse disorders [15, 22–24]. Particularly in the context of a pandemic, people who score high on the neuroticism trait are more likely to experience more stress, not only in response to the threat of the disease, but also in reaction to social restrictions [25]. Recent studies during COVID_19 pandemic has also shown that individuals who score higher on neuroticism experienced more generalized anxiety and depressive symptoms [26], more worrying and negative affect in their daily lives [25, 27], found work/study restrictions and hygiene measures more restricting and experienced lower subjective well-being [28], had more concerns about finances and relationships, and were less optimistic about the duration estimates related to the COVID‐19 pandemic [29]. Also, some researchers proposed that it is probable that people who score high in the neuroticism trait might concentrate more on COVID-19-related information and pandemic consequences more than people who score lower in this trait [11]. As a result, those high in neuroticism can adopt social distancing to avoid COVID-19 infection [30, 31], which can lead to experiencing even more mental health issues.

Extroversion is another trait that has shown strong correlations with mental health outcomes. As introverts and extroverts demonstrate substantially different attitudes toward social life [32, 33], the effects of social distancing might vary depending on individuals’ extroversion levels. Individuals who score high in extroversion tend to experience positive emotions, activity, assertiveness, need for stimulation, and gregariousness compared to people with low on extroversion [34, 35]. Individuals who score higher in extroversion trait are energized and flourish by being around other people, and enjoy activities involving social interactions which is helpful in increasing their level of positive emotions [36]. Higher score in extroversion trait has been associated with better perceived health [37], well-being [21, 38–40], resilience [41, 42], positive affect [43, 44], and positive mental health [45]. In contrasts, individuals who score high in introversion typically tend to have fewer social interactions than extroverts, experience more psychological problems in general, experience more intense emotions and more struggles in regulating their emotions, and have more adjustment problems [43, 46, 47].

There is controversy regarding the association between the extroversion trait and mental health during a pandemic. One group of researchers claim that the lifestyle associated with social distancing during the pandemic might feel more unusual to extroverts than to people who score lower in extroversion, as individuals who score higher in extroversion trait are energized by crowds and social interactions, and seek the company of others when stressed [48]. Therefore, social isolation by inhibiting their need for social engagement might have more deleterious effects on the mental health of individuals who score higher in extroversion compared to people who score lower in extroversion, who continue to enjoy spending time in their solitude like the time before social distancing [49]. Many articles published on widely-visited popular websites such as Bloomberg (2020), Reuters (2020), The Conversation (2020) and the psychology website Psychology Today (2020) have introduced introversion as an asset for flourishing during social isolation related to COVID-19 [50–53]. Conversely, some researchers proposed that extroverts are more capable of adjusting to life-changing events, experience more positive affect, and keep their positive affect longer than people who score lower in extroversion, especially in more emotionally distressing situations [39]. Moreover, extroverts usually have stronger social relationships which might have a buffering effect during the pandemic crisis [54]. For instance, compared to people who score lower in extroversion, people who score higher in extroversion have more extended social networks [55, 56], consequently more support would be available to them during a difficult time. They also have higher quality relationships [55], experience more friendships satisfaction [57], and perceive their social support higher [58, 59]. Therefore, it is possible they only experience small declines in their mental health compared to people who score lower in extroversion. Some recent research findings during COVID-19 pandemic confirmed this possibility, showing negative outcomes for people who score lower in extroversion and more positive outcomes for individuals who score higher in extroversion. Studies have found negative associations between higher extroversion and loneliness, anxiety, and depression experienced as a result of COVID-19 changes [60, 61], and associations between experiencing more optimism, psychological adjustment and optimal functioning such as creative thinking with extroversion [29, 62].

## The present study

For this study, online questionnaires were sent to adults living across Canada using Qualtrics, a survey platform, distributed through social networks and from the email listings of the University of Alberta. Before participating in the study, all participants gave their consent through Qualtrics in a question asking “Do you wish to continue the survey, if you do your consent to participate is implied, with two options of “I consent” and “I do not wish to continue”. This study was reviewed for its adherence to ethical guidelines by a Research Ethics Board at the University of Alberta (Pro00102974). Participation in the study was voluntary and ten random participants received a $50 gift card of their choice.

The primary aim of this study was to examine how personality traits of neuroticism and extroversion are related to the mental health of Canadians during the COVID-19 pandemic. As many previous studies have shown associations between personality traits and mental health [45], it is important to investigate how personality traits are associated with mental health during the Covid-19 pandemic, in which “new normal” is introduced due to imposed social and public life restrictions. Based on the literature presented above we hypothesize that the mental health status of Canadians during the COVID-19 epidemic is associated with personality traits of neuroticism and extroversion. We hypothesized that people who score higher in neuroticism are experiencing more difficulties adjusting to the new situation, therefore experiencing more mental health issues, as neuroticism is associated with increased vulnerability to stress and changes in life circumstances. Our expectations regarding extroversion were less clear. On the one hand, the lifestyle of social distancing during the pandemic might feel more difficult to people who score higher in extroversion than to people who score lower in extroversion, as people who score higher in extroversion seek the company of others when stressed [48], therefore social isolation could have more deleterious effects on their mental health compared to people who score lower in extroversion. On the other hand, many studies have indicated that people who score higher in extroversion perform better in adjusting to life changing events, showing more positive affect, and keeping their positive affect longer than people who score lower in extroversion, especially in more emotionally ambivalent situations [39]. This study contributes to our understanding the relationship between personality traits of neuroticism and extroversion, and mental health of Canadians during the COVID-19 pandemic. In particular, as there are various predictions regarding the effects of the pandemic (such as social isolation) on mental health among extroverts, this study could be enlightening.

We also hypothesized that both demographic characteristics specific to the COVID-19 situation (age, gender, and job status) and the ones specific to the COVID-19 situation (change in income, domestic conflict, social interaction loss and, child care loss variables) contribute to the mental health of Canadians.

To assess mental health, we examined the relation of the personality traits of neuroticism and extroversion to emotional, psychological and social well-being. We hypothesized that neuroticism and extroversion have stronger associations with emotional well-being. Also, we expected associations with psychological and social well-being, as the new circumstances related to the COVID-19 pandemic had imposed many social restrictions on people’s lives which is detrimental to their social well-being and also limited the opportunities for people to experience self-acceptance, personal growth, autonomy, purpose in life, a sense of mastery, and positive relations with important others which is how Ryff (1989) defines psychological mental health [63].

## Materials and methods

### Participants

A sample of 1429 initiated our survey, after removing the incomplete data, 1096 participants (880 females, 202 males, and 14 other genders; mean age = 26.47 years [SD = 9.5; range 18 to 86 years]) were recruited for this study who completed a battery of questionnaires during June and July 2020. Participants were required to: (1) be at least 18 years of age; (2) reside in Canada; and (3) consent to participate.

### Measures

#### Mental health

The short form of the Mental Health Continuum (MHC-SF) is derived from the long-form (MHC-LF) and consists of 14 items representing theoretically derived feelings of well-being (MHC-SF; Keyes et al., 2008; Lamers et al., 2012). Participants rated the frequency of each feeling during the past month on a 6-point Likert scale from never (1) to every day (6), which means that the total score on the scale could range from 14 to 84 points. MHC-SF is a multidimensional test and contains three items representing emotional well-being (eg., during the past month, how often did you feel interested in life), six items representing psychological well-being (one item from each of the 6 dimensions of Ryff’s (1989) [63] model of psychological well-being; eg., during the past month, how often did you feel that you had warm and trusting relationships with others), and five items represent social well-being (one item from each of the 5 dimensions of Keyes’ (1998) model of social well-being; eg., during the past month, how often did you feel that you had something important to contribute to society) [64]. Emotional well-being represents a balance of positive over negative emotions and the existence of life satisfaction, following the hedonic tradition in this area of research [21]. Psychological well-being is in accordance with the eudaimonic tradition in well-being and defines people as mentally healthy when they experience self-acceptance, personal growth, autonomy, purpose in life, a sense of mastery, and positive relations with important others [63]. Social well-being, also in accordance with the eudaimonic tradition. According to this approach, optimal functioning in a community is vital for understanding effective functioning and well-being [65] and individuals are defined as mentally healthy when they feel that they fit in and contribute to the society, so that they understand how society operates, and acknowledge that society develops in a positive direction [65]. Together, emotional, psychological, and social wellbeing construct the definition of positive mental health which in line with the definition of the World Health Organization (2005) defining mental health as when an individual experience feeling of well-being (emotional well-being), and when functioning effectively in both private (psychological well-being) and social life (social well-being) [45, 66].

We computed a mean score, with higher scores indicating higher levels of emotional well-being, psychological well-being, and social well-being. In the current study, Cronbach’s alpha was .86 for emotional well-being, .81 for social well-being, and .86 for psychological well-being.

#### Extroversion and neuroticism traits

We used the two extroversion and neuroticism scales from The Big Five Inventory-2 Short (BFI-2-S)22 which is a self-report measure of personality traits based on the FFM and is based on the most commonly used model of personality [12]. Extroversion (e.g. “I don’t mind being the center of attention”) and neuroticism (e.g. “I am easily disturbed”) consist of 10 items that are answered on a 5-point Likert scale, where 1 = Disagree, 3 = Neutral, and 5 = Agree. A total score was computed for each personality trait (10–50), with higher scores showing higher levels of that personality trait. Cronbach’s alpha in the current study was .92 for extroversion and .89 for neuroticism.

### Demographics

The following demographic characteristics of all participants were collected: 1) age, 2) gender, 3) their current job status and if there has been a change in their income over the last 2 months, 4) pre-existing mental health issues. We believe the mentioned characteristics could have important contributions to the well-being of participants as some of these characteristics had changed as a result of COVID-19 pandemic. For example, many Canadians lost their job and income as a result of the pandemic that could impact their well-being [67]. Also, age and gender have shown to make significant differences in the mental health status of people during COVID-19 pandemic [68, 69]. Also, we asked our participants some specific questions regarding their personal experience with COVID-19 pandemic such as, if they or anyone living in their household were diagnosed with COVID-19, if they experienced any kind of domestic conflicts as a result of pandemic and staying more at home, to what extent the pandemic has affected their social interactions, and if losing childcare services affected them in some way. We believe the people’s experiences with COVID-19 pandemic could impact their mental health. Further demographic information is provided in Table 1.

**Table 1:** Sample demographics characteristics.

Demographics	Options	(%)
*Current Job status*		
	Not employed	16.7%
	Temporary/ Part-time Employment	20.9%
	Full-time Employment	21.5%
	Pursuing Further Studies (student)	40.9%
*Change in income*		
	Decrease in income	35.2%
	Increase in income	21.4%
	No change	43.3%
*COVID-19 diagnosis*		
	The person him/herself	.2%
	Someone in their household	.5%
	No one	99.3%
*Experience of domestic conflict*		
	Yes	25.1%
	No	74.9%
*Experience of childcare service loss*		
	Yes	5.3%
	No	94.7%
*Social interaction loss*		
	It has not affected my social interactions.	2.3%
	It has somewhat affected my social interactions.	33.5%
	It has largely affected my social interactions.	64.3%
*Prior mental health issues*		
	Yes	22.5%
	No	77.5%

## Analyses

All analyses described as follows were conducted with SPSS (Version 26). To study the association of personality traits of neuroticism and extroversion with positive mental health, we first computed Pearson correlation coefficients between neuroticism and extroversion on the one hand, and emotional, psychological and social well-being on the other hand, as well as the demographic characteristics. Then, hierarchical multivariate regression models were used to assess the relationship between independent variables and the outcome variable. Those demographic characteristics with significant association to the dependent variables (emotional, psychological, and social well-being) during bivariate analyses were entered into the hierarchical regression models (the first and the second models).

In the regression model, gender, age, prior mental health history, and job status were entered in the first block in order to control for potential confounding variables (the final block of the three hierarchical regression analyses is shown in Table 3). We chose to enter these variables first because these demographic variables were not specific to the COVID-19 situation (controls). In the second block, change in income, domestic conflict, social interaction loss and, child care loss variables (variables specific to the COVID-19 situation) were entered into the model. Finally, after controlling for demographic variables independent of COVID-19 situation and specific to COVID-19 situation, personality traits of neuroticism and extroversion were entered into the model.

## Results

Table 2 shows the means, standard deviations, and correlations between neuroticism and extroversion personality traits, positive mental health subscales. The neuroticism and extroversion personality traits were significantly and negatively correlated at r(1095) = -.19. Also, neuroticism and extroversion were significantly related to positive mental health subscales.

**Table 2:** Means, standard deviations and correlations between positive mental health, demographics characteristics and neuroticism and extroversion personality traits.

	Demographics															Well-being			Personality	
		1	2	3	4			5		6		7	8		9	10	11	12	13	14
	Mean (SD)	-	2.1	3.2	4.1	4.1	4.3	5.1	5.2	6.1	6.2	7.1	8.1	8.2	-	-	-	-	-	-
1-Age	26.47 (9.35)	-																		
2-Gender 2.1-Female 2.2-Male	-	-.10** REF	-																	
3-Mental health 3.1-Yes 3.2-No	-	-.06* REF	.08**	-																
4-Job status 4.1-Full-time 4.2-Part-time 4.3-Student No job	-	.35** -.11** -.17** REF	-.02 .06** -.04	-.07 -.00 .07*	-															
5-Income change 5.1-Increase 5.2-Decrease No	-	-.18** .04 REF	.03 .05	.01 .04	-.09** -.04	-														
6-Covid diagnosed 6.1-The person 6.2-Household No one	-	.02 .05 REF	-.03 -.25	-.02 -.04	-.02 .01	.03 -.00	-.02 .02	-.02 -.00	.01 -.00	-	-									
7-Domestic conflict 7.1Yes 7.2 No	-	-.12** REF	.01	.10**	-.02	.05	-.02	.00	.05	-.02	.10**	-								
8-social interaction 8.1-Somewhat 8.2-Largely No change	-	.02 -.01 REF	-.03 .05	-.04 .05	-.01 .03	.00 -.01	.04 -.04	.08** -.06	-.07* .07*	-.03 .03	.05 -.04	-.02 -.02	-	-						
9-childcare loss 9.1-Yes 9.2-No	-	.15** REF	-.00	.00	-.08**	-.01	.07*	.00	.03	-.01	.03	.06*	.02	-.01	-					
10-Emotional WB	9.32 (3.25)	.08**	.04	-.15**	-.03	-.02	.10**	.05	-.10**	.00	-.04	-.20**	.13**	-.15**	-.03	-				
11-Psychological WB	17.68 (6.25)	.12**	-.02	-.17**	.11**	-.04	.11**	.01	-.08**	.02	-.01	-.20**	.14**	-.16**	-.05	-.05*	-			
12-Social WB	16.91 (8.47)	.17**	-.06*	-.16**	-.02	-.07*	.16**	-.00	-.07**	.04	.01	-.20**	.12**	-12**	-.03	.58**	.67**	-		
13-Neuroticism	16.91 (8.47)	.19**	.20**	.33**	.05*	.03	-.12**	.02	.08**	-.01	-.01	.17**	.08**	-.01	.04	-39**	-.41**	-.46**	-	
14-Extroversion	19.20 (9.12)	.05	-.00	-.00	-.03	-.02	.07*	.02	.01	-.00	-.00	.02	-.07*	.05	.03	.20**	.28**	.35**	-.19**	-

WB: well-being.

* p < .005

** p < .001.

In agreement with our expectations, neuroticism was negatively and significantly related to all subscales of emotional and psychological and social well-being when controlling for demographics, and the extroversion trait (see Table 3). Moreover, extroversion was also positively and significantly related to all three scales of emotional well-being, psychological and social well-being (see Table 3). On top of the demographics, the two personality traits on top of the demographic characteristics, explained 22% of emotional well-being, 33% of psychological well-being, and 26% of the social well-being of the variance. These results showed neuroticism and extroversion personality traits were both related to all the three subscales of mental health.

**Table 3:** Hierarchical regression analysis (standardized beta weights) of personality traits of neuroticism and extroversion in relation to emotional, psychological and social well-being, controlled for demographics.

	Social well-being			Emotional well-being			Psychological well-being		
	Beta	95% CI		Beta	95% CI		Beta	95% CI	
**Age**	.06*	.00	.07	-.01	-.02	.01	.02	-.02	.05
**Gender**				N/A	N/A	N/A	N/A	N/A	N/A
Female	.01	-.51	.98						
Male	REF								
**Prior mental health issues**									
Yes	-.03	-1.18	.29	-02	-.61	.26	-.01	-1.10	.52
No	REF			REF					
**Job status**									
Full time	.06*	.11	1.68	.04	-.12	.77	.03	-.36	1.36
Part time	-.02	-1.11	.37	N/A	N/A	N/A	N/A	N/A	N/A
No	REF			REF					
**Change in income**									
Decrease in income	-.04	-1.10	-.12	-.05*	-.75	-.02	-.02	-.99	.36
No change	REF			REF			REF		
**Domestic conflict**									
Yes	-.13**	-2.45	-1.09	-.15**	-1.52	-.71	-.13**	-2.80	-1.31
No	REF			REF			REF		
**Social interaction loss**									
Somewhat affected	.04	-1.41	2.55	-.10	-1.89	.45	-.05	-2.87	1.50
Largely affected	-.06	-2.71	1.22	-.23**	-2.73	-.40	-.19*	-4.81	-.48
Not affected	REF								
**Child Care**	N/A	N/A	N/A	N/A	N/A	N/A			
**Yes**							-.04	-2.86	.02
**No**							REF		
Neuroticism	-.30**	-.24	-.16	-.30**	-.13	-.09	-.34**	-.30	-.22
**Extroversion**	.23**	.11	.18	.16**	.04	.08	.30**	.18	.25

N/A: not applicable.

Note. Emotional well-being: R^**2**^ = .02 for block 1 (F(3, 1094) = 16.20; p < .001); ΔR^**2**^ = .06 for Block 2 (Fchange(8, 1094) = 16.01; p < .001). ΔR^**2**^ = .12 for Block 3 (Fchange(2, 1094) = 86.10; p < .001). Psychological well-being: R^**2**^ = .04 for block 1 (F(2, 1094) = 23.00; p < .001); ΔR^**2**^ = .06 for Block 2 (Fchange(5, 1094) = 10.91; p < .001); ΔR^**2**^ = .22 for Block 3 (Fchange(2, 1094) = 186.53; p < .001). Social well-being: R^**2**^ = .05 for block 1 (F(3, 1094) = 22.59; p < .001); ΔR^**2**^ = .05 for Block 2 (Fchange(8, 1094) = 11.25; p < .001); ΔR^**2**^ = .16 for Block 3 (Fchange(2, 1094) = 113.79; p < .001).

* p < .005

** p < .001.

## Discussion

This study examined how personality traits of neuroticism and extroversion (using the Five-Factor Model (FFM) as our framework) are related to the current mental health of Canadians (emotional, psychological and social well-being) during COVID-19 pandemic. Our main finding was that personality traits of neuroticism and extroversion are associated with the current mental health of Canadians during COVID-19 pandemic, with extroversion positively related to the three subscales and neuroticism negatively related to the three subscales. The two measured personality traits, on top of the demographics, explained 22% of emotional well-being, 33% of psychological well-being, and 26% of the social well-being of the variance. In support of our hypothesis, both neuroticism and extroversion were related to all three subscales of positive mental health, with stronger association with psychological and social well-being.

Also, our results show that demographic characteristics specific to COVID-19 pandemic have impacted mental health status of Canadians. For instance, age was positively correlated with the emotional, psychological and social well-being of our participants. This is in line with previous studies showing the association between age and mental health during COVID-19 pandemic [69, 70]. Also, the existence of domestic conflicts negatively affected the emotional, psychological and social well-being of our participants. This is also in line with previous studies during COVID-19 pandemic indicating that domestic conflicts during the pandemic (such as have resulted in deteriorated mental health [71].

### Neuroticism

In line with our expectations, neuroticism is negatively and significantly associated with emotional, psychological and social well-being. Our findings are in line with earlier studies indicating that neuroticism is associated with more mental health issues in general [15, 21–23] and also with recent studies during COVID-19 pandemic showing that individuals who score higher on neuroticism are experiencing more mental health problems [25, 27–29]. According to previous research individuals who score high on the neuroticism trait often experience more negative affectivity and respond worse to stressors that leave them susceptible to unfavorable outcomes in the context of a stressful experience such as COVID-19 pandemic [18], predisposing them to psychological distress [19]. High neuroticism is associated with a range of detrimental health outcomes, including lower subjective well-being [63], depressive symptoms, anxiety, mood, and substance use disorders [15, 22, 23].

### Extroversion

Overall, higher extroversion was associated with higher emotional, psychological and social well-being. The finding that extroverts are experiencing less mental health issues as a result of social distancing and lockdown measures is in line with previous studies demonstrating that extroversion is associated with better mental health in general [19, 45, 72] and during COVID-19 pandemic [60, 61]. A possible explanation could be that according to previous research, compared to people who score lower in extroversion, extroverts are more capable of adjusting to life changing events, such as the new circumstances the world is currently experiencing as they use adaptive strategies such as reappraisal, problem solving, or acceptance more than people who score lower in extroversion [73]. Individuals who score higher in extroversion also experience more positive affect, and keep their positive affect longer, especially in more emotionally stressful situations [39], are less susceptible for mental illnesses [23, 74] and are generally happier [38, 39]. Moreover, as people who score higher in extroversion have more friends and social networks, higher perceived social support, and higher quality relationships [55, 56, 58, 59], they may rely on their social support to maintain their positive mental health.

## Limitations and future directions

The current study has some limitations that need to be considered. First, while the current study covered a wide age range, the sample was not completely representative of the general population, as around 41% of our participants were students with a mean age of 26.47 years and about 80% were female. The second limitation of this study is that although we aimed for participants from all over Canada, most of our sample reside in Alberta (the place the study was conducted). The third limitation of this study is that our data were gathered only at one point in time during the first wave of COVID-19 and as it was summer, people could spend more time outside. At this time, the participants had experienced few (if any) effects of COVID-19 (see Table 1). For example, 99% of the participants neither themselves were diagnosed with COVID-19, nor anyone in their household were. The effects of personality on mental health could change as the personal relevance changes. Another limitation of the present study is that the measurements used to assess personality and well-being were the short version of the measurements. We chose the short version of tests in order to collect data from a large group of participants who completed the tests online. Around 79% of people who started answering the questions answered all the questions and we believe if we used the longer versions, a lot of our participants wouldn’t have finished all the questions. However, we made sure our measurements had acceptable psychometric properties, for instance MHC-SF had shown great internal consistency (> .80) and discriminant validity in different countries [45, 64]. Also, The Big Five Inventory-2 Short (BFI-2-S)22 have been found to be useful for examining facet-level traits in reasonably large samples and it is estimated that they retain approximately 85% of the full BFI-2 facet scales’ reliability and validity [75].

Regardless of these limitations, the current study makes important contributions to understanding the associations between personality traits of neuroticism and extroversion, and mental health of Canadians during a global health crisis and could lead to a revelatory direction for future research into personality and mental health. Future research should investigate the impacts of personality traits of neuroticism and extroversion on mental health of Canadians during the second wave of COVID-19 in winter when people had to stay at home more than in summer due to the cold weather. Also investigating the impacts of other personality traits on mental health could be informative and help public health services provide personality-appropriate mental health services during this pandemic.

## Conclusion

These findings, taken together, confirm previous research findings demonstrating neuroticism and extroversion are impactful on mental health [47]. Our results indicated that individuals who scored higher on neuroticism were experiencing more mental health issues and in contrast individuals who scored higher in extroversion were experiencing less mental health issues. These relationships are similar to the pre-pandemic times, we believe the reason that the pandemic has not changed the relationship between the extroversion trait and mental health is that people who score higher in extroversion generally experience more positive affect, and keep their positive affect longer, especially in more emotionally stressful situations [39], they also have more friends and social networks, higher perceived social support, and higher quality relationships [55, 56, 58, 59], they may rely on their social support to maintain their positive mental health. Moreover, we believe that the reason the reason that the pandemic has not changed the relationship between the neuroticism trait and mental health is that neuroticism is associated with more mental health issues in general [15, 21–23].

This study has important implications for understanding how different individuals are experiencing the pandemic. Public health messages can be tailored to consider these differences. Also, since individuals have had different mental health effects during the pandemic, they may also cope with the post-pandemic world differently.
